# Implementing Supported Digital Enhanced Cognitive Behavior Therapy for Binge Eating Disorder in Routine Care: Mixed Methods Service Evaluation

**DOI:** 10.2196/92069

**Published:** 2026-07-17

**Authors:** Emma L Osborne, John Powell, Chloe Brown, Gemma Cresswell-Nash, Lisa Debrou, Emmanuel Defever, Susie Greenwood, Jade Horton, Emily Hunter, Amanda Lees, Maddison Moore, Ciarán Newell, Becca Randell, Claire Rosten, Natasha Shaw, Vivi Yao, Rebecca Murphy

**Affiliations:** 1 Centre for Research on Eating Disorders at Oxford Department of Psychiatry University of Oxford Oxford, England United Kingdom; 2 Nuffield Department of Primary Care Health Sciences University of Oxford Oxford United Kingdom; 3 Kent & Medway All Age Eating Disorders Service North East London Foundation Trust NHS Maidstone United Kingdom; 4 Credo Therapies Oxford United Kingdom; 5 Health Innovation Wessex Southampton United Kingdom; 6 Health Innovation Kent Surrey Sussex Worthing United Kingdom; 7 School of Psychology University of Sussex Falmer United Kingdom; 8 Health Innovation West of England Bristol United Kingdom

**Keywords:** binge eating disorder, CBT-E, digital intervention, enhanced cognitive behavior therapy, guided self-help, implementation, mixed methods, National Health Service, NHS, routine care, service evaluation

## Abstract

**Background:**

Binge eating disorder (BED) is highly prevalent and impairing; yet, the UK national guideline–recommended first-line treatment of guided self-help (ie, supported program-led interventions in which content is delivered by the program with brief support) remains underused in routine National Health Service (NHS) care. Digital delivery offers a scalable approach, but evidence from real-world NHS settings is limited.

**Objective:**

This real-world evaluation aimed to pilot a supported digital program-led version of enhanced cognitive behavior therapy (CBT-E) in a specialist adult eating disorder outpatient service within the UK NHS and assess its implementation in routine practice, acceptability, and preliminary clinical outcomes.

**Methods:**

An independent evaluation service conducted this service evaluation in a specialist NHS eating disorder service. Adults assessed by NHS clinicians as having features consistent with BED and for whom a supported program-led intervention was considered appropriate were offered a 12-session digital CBT-E program (delivered over 8-12 weeks), with brief remote support from trained nonspecialist practitioners. Patients completed an in-program suitability assessment to ensure that the intervention was appropriate (eg, excluding those with suicidal ideation). Self-report measures of binge eating frequency, eating disorder psychopathology, secondary impairment, and depressive symptoms were collected before and after the program. Outcomes were analyzed using paired *t* tests. All patients were offered an optional interview. Semistructured interviews with 8 patients, 2 supporters, and a survey completed by 6 staff members were analyzed thematically to explore experiences of the intervention within the care pathway.

**Results:**

Between January and October 2024, 43 patients registered, and the program was deemed suitable for 36 (84%), all of whom completed preprogram assessments. Among these, 6 were still using the program at the end of the evaluation period, and 30 had either completed or discontinued the program. These patients (n=30) completed a mean of 8 of 12 sessions; 16 completed the full program and postprogram assessments. Among these 16, significant reductions were observed in binge eating frequency (mean 18.91, SD 11.28 to 1.88, SD 1.82; *P*<.001) and eating disorder psychopathology (mean 3.94, SD 1.20 to 1.85, SD 1.14; *P*<.001), with similar improvements in secondary impairment and depressive symptoms (both *P*<.001). Qualitative feedback highlighted the program’s accessibility, its role in keeping patients on track, and its positive impact on staff development and satisfaction. Some patients initially expressed skepticism about the digital format, and staff noted challenges for individuals with low motivation or co-occurring conditions.

**Conclusions:**

This evaluation suggests that the supported digital program-led version of CBT-E can be successfully implemented within a specialist NHS service and may lead to improvements in clinical outcomes. This approach may increase access to guideline-recommended first-line treatment for BED in routine care. Further research with larger samples and controlled designs is needed to confirm effectiveness and evaluate resource use and cost-effectiveness.

## Introduction

Binge eating disorder (BED) is the most common eating disorder globally, with an estimated lifetime prevalence of approximately 2.8% in women and 1% in men worldwide [1]. It is characterized by recurrent binge eating episodes, defined as consuming unusually large amounts of food with a distressing sense of loss of control, occurring at least once a week for at least 3 months [2]. BED is associated with significant physical and psychological impairment, reduced quality of life, and substantial societal costs [3-6]. In the United Kingdom, eating disorders involving binge eating are estimated to cost the economy more than GBP £3.5 billion (GBP £1=US $1.32 as of June 26, 2026) annually [6], underscoring the need for accessible and effective treatment.

The latest UK National Institute for Health and Care Excellence (NICE) guidelines recommend BED-focused guided self-help (GSH) as the first-line treatment for adults with BED [7]. Within the UK National Health Service (NHS) care pathway, individuals with suspected eating disorders are typically referred from primary care to specialist community-based eating disorder services, where GSH may be delivered following assessment as part of a stepped-care approach. GSH can be conceptualized as a supported program-led intervention in which therapeutic content is delivered through structured materials, with brief support focused on facilitating engagement [8,9]. In this paper, we use the term “supported program-led” when referring to the intervention evaluated and “GSH” when referring to guideline recommendations and the broader literature. According to NICE guidance, GSH should use cognitive behavioral materials and be supplemented by brief supportive sessions, typically 4 to 9 sessions of around 20 minutes over a 16-week period. More intensive interventions, such as group or individual eating-disorder–focused cognitive behavioral therapy (CBT-ED), should be offered if GSH is ineffective after several weeks.

Meta-analytic evidence supports the effectiveness of GSH, particularly programs based on printed cognitive behavioral materials, in reducing eating disorder psychopathology and binge eating, with the strongest effects observed in individuals with BED [10]. More recent studies have examined digital formats, reporting favorable outcomes and highlighting potential advantages in scalability, accessibility, and cost-effectiveness [11,12]. However, most existing studies have been conducted in controlled research settings, with limited evaluation of implementation within routine health care services, particularly in the UK NHS context.

Despite strong recommendations and a robust evidence base, implementation of GSH for BED remains inconsistent within NHS services. Current data indicate that only 62% of adult teams and 67% of all-age teams offer GSH [13], highlighting a significant shortfall in the provision of guideline-recommended care.

One UK-based service evaluation of virtually delivered GSH for BED and bulimia nervosa found the approach to be feasible, acceptable, and associated with reductions in eating disorder symptoms [14]. However, the intervention relied on a self-help book and relatively intensive guidance from coaches (more than 5 hours per patient), exceeding the brief support model recommended by NICE. Fully digital programs offer the potential to reach larger populations than printed self-help materials and may provide additional advantages in terms of engagement, personalization, and discretion, particularly given the shame and stigma often associated with binge eating. At the same time, there is a need to ensure that GSH is delivered in a manner consistent with NICE guidance, which recommends brief, low-intensity support (typically between 80 and 180 minutes in total across all sessions). To date, no studies appear to have evaluated the implementation, acceptability, and effectiveness of fully digital GSH for BED delivered in a way that is consistent with NICE guidance in routine clinical practice.

The development of a digitally delivered intervention supports the NHS 10 Year Health Plan, which emphasizes the transition from analog to digitally enabled services to improve access and efficiency [15]. In this context, we piloted a supported digital program-led version of enhanced cognitive behavior therapy (CBT-E), a leading approach within CBT-ED, adapted from both its therapist-led [16] and printed self-help [17] formats. The printed program, *Overcoming Binge Eating* [17], is one of the most recognized GSH interventions and has demonstrated effectiveness in reducing binge eating [10,18]. The digital program was designed to build on this by incorporating features such as interactivity, personalization, and automated reminders to enhance engagement and scalability. It allows individuals to work through the program independently (pure self-help) or with brief support from a nonspecialist (supported delivery), supporting scalable implementation within routine care.

The pilot took place in a specialist eating disorder service in the UK NHS and was designed to improve local service delivery. Adults with features consistent with BED were offered the intervention following clinical assessment and used the program alongside brief support calls with a staff member within the NHS service. This evaluation aimed to assess whether the intervention could be implemented in routine NHS care, the extent of treatment uptake and completion, its acceptability to patients and staff, and whether patients who completed the program showed improvements in clinical outcomes.

## Methods

### Ethical Considerations

This project was conducted as a service improvement initiative within an NHS service and did not constitute research as defined by the UK Health Research Authority. According to Health Research Authority guidance, service evaluations designed to assess and improve local service delivery do not require review by a research ethics committee [19]. This project met these criteria, because it evaluated a service implemented within routine care without randomization or deviation from standard treatment pathways.

The project was reviewed and approved through local NHS governance processes within the participating service. Quantitative data were collected as part of routine care via the digital program, and patients were informed within the program about how their data might be used. Informed consent was obtained for the interviews and staff survey, because these activities were outside routine care. All data were anonymized in accordance with NHS data protection and suppression guidelines.

### Design

This digital transformation was a service improvement project designed to enhance the quality of care within an existing health care service. The evaluation was conducted by an independent evaluation service, Health Innovation Wessex, and a full evaluation report is available [20]. The independent evaluation service conducted analyses of clinical outcomes and collected and analyzed all qualitative data, including interviews and the staff survey. The first author (ELO) conducted descriptive analyses of program uptake and usage, including suitability, completion rates, and patient satisfaction. All analyses and interpretations were reviewed collaboratively by all authors. No authors with commercial interests were involved in the analysis or interpretation of the clinical outcome data.

A pragmatic convergent mixed methods design was used, in which quantitative data (including clinical self-report measures, program usage and completion data, and patient-reported satisfaction) and qualitative data (semistructured interviews and an open-ended staff survey) were collected in parallel and brought together at the interpretation stage.

### Setting

The All-Age Eating Disorder Service for Kent and Medway is a specialist eating disorder service within North East London NHS Foundation Trust. It provides evidence-based outpatient treatment to individuals in Kent, South East England.

### Patients and Procedure

Patients entered the pilot through standard care pathways within the NHS eating disorder service, either through referral or self-referral. No additional recruitment procedures were used.

Patients underwent a clinical assessment by interview with trained clinicians within the NHS eating disorder service (eg, clinical psychologists or other clinicians with expertise in assessing eating disorders) to determine whether the supported program-led intervention was appropriate and safe. This assessment formed part of routine care and involved clinical judgment regarding diagnosis, risk, and the patient’s likely ability to engage with a supported program-led intervention. The criteria used for this assessment were defined by the NHS eating disorder service. Adults with BED who could safely engage with the intervention were offered it between January and October 2024.

The NHS eating disorder service had previously established a GSH care pathway based on the printed program *Overcoming Binge Eating*. This service improvement project evaluated the delivery of the intervention in a digital format within this existing care pathway.

Patients who accepted the offer registered for the program and completed a digital in-program suitability assessment (see the Program Suitability Questionnaire subsection). Patients for whom the program was deemed unsuitable at this stage, or who chose not to proceed, were offered treatment in line with routine care pathways, including the existing GSH pathway based on the printed program. Individuals requiring more intensive or specialist input were excluded and offered alternative treatment within the service. As this assessment was conducted within routine care, data on the number of patients who declined the program when it was offered and their subsequent treatment pathways were not systematically recorded. Those for whom the program was considered suitable proceeded with the digital program alongside support.

### Staff Training and Supervision

A clinical psychologist and a researcher from Credo Therapies, an impact-driven company with an exclusive license for the digital program-led version of CBT-E, delivered two 3-hour live online training sessions to the NHS eating disorder service staff involved in the pilot. These included the 2 mental health and well-being practitioners (MHWPs) who delivered the support. The training covered GSH for eating disorders, the digital program, the role of the supporter, and the structure and aims of the support sessions. MHWPs are NHS-employed practitioners trained to deliver brief, structured psychological interventions for people with common mental health problems, typically within NHS Talking Therapies and similar settings. They commonly hold undergraduate degrees in psychology or related fields and receive additional training in delivering low-intensity, evidence-based interventions. In other services, GSH may be delivered by different trained staff, such as assistant psychologists or support workers.

Credo Therapies also provided monthly 1-hour clinical supervision sessions to both supporters during the pilot. Supervision was delivered by a clinical psychologist and a researcher with expertise in CBT-E and eating disorders and experience in delivering and supervising GSH. The training and supervision were designed to ensure consistent delivery of the support component, maintain fidelity to the supporter role, and enhance supporter confidence.

### Intervention

The intervention was the supported digital program-led version of CBT-E. CBT-E is an empirically supported, therapist-led treatment for eating disorders [16]. The program is a psychological treatment for people who experience recurrent binge eating. It is a CBT-ED program delivered via a smartphone app and website. The program consists of 12 sessions (typically taking around 10-20 minutes to complete) delivered over approximately 8-12 weeks. Sessions were made available at fixed time intervals, initially twice weekly, with later sessions spaced farther apart (up to 2 weeks). Content was presented primarily in text and figure format and included interactive components such as a real-time self-monitoring tool and task lists. In this evaluation, the program was piloted with external support in the form of brief virtual sessions delivered by MHWPs who were familiar with the program. The purpose of these sessions was to help patients follow the program by offering encouragement, clarification, space for reflection, and accountability rather than providing clinical advice or therapy. A handbook was provided to guide MHWPs in delivering support, including recommended session lengths (10-20 minutes) and guidance to ensure consistency with NICE guidelines for GSH.

### Quantitative Methods

#### Program Suitability Questionnaire

Following the initial clinical assessment described above, a second-stage in-program suitability questionnaire was completed immediately before starting the intervention. This acted as an additional safeguarding step to identify any changes in risk or circumstances between the service-led assessment and program initiation, ensuring that the intervention remained appropriate. As this was an initial pilot, the NHS eating disorder service determined that the intervention would be offered only to individuals with BED.

The within-program suitability criteria, also defined by the NHS eating disorder service, ensured that the program was offered only to individuals whose needs could be safely and appropriately met by the supported digital program-led intervention. The in-program suitability questionnaire included items assessing key safety and suitability criteria (eg, risk of harm, presence of binge eating, and absence of compensatory behaviors) and complemented, rather than replaced, clinician judgment. It also included demographic items, which were not used to determine program suitability.

The program was considered unsuitable for individuals experiencing suicidal ideation or self-harm and those with significant physical health risks, as these presentations were considered to require a higher level of care than could be provided within this intervention. For these individuals, automated feedback explained the reasons and redirected them to alternative support options. These feedback messages were also developed by the NHS eating disorder service.

#### Clinical Outcomes

Three standardized self-report measures were integrated into the program at the start (preprogram) and end (postprogram) to assess key clinical domains relevant to BED and associated impairment.

Eating disorder features over the past 28 days were assessed using the Eating Disorder Examination–Questionnaire (EDE-Q) [21], a widely used and validated measure of eating disorder psychopathology. The EDE-Q was modified within the digital program to also capture subjective binge eating (Multimedia Appendix 1). However, for the purposes of this evaluation, analyses focused on objective binge eating episodes and global EDE-Q scores. Two outcome variables were derived: a single item assessing the number of objective binge eating episodes experienced, selected because of its direct relevance to the program’s primary target of reducing binge eating, and the severity of general eating disorder psychopathology, measured by the global EDE-Q score, where higher scores indicate greater severity (range 0-6). Secondary impairment due to eating disorder features was assessed using the Clinical Impairment Assessment [22], a widely used and validated measure of psychosocial impairment associated with eating disorder symptoms, where higher scores indicate greater impairment (range 0-48). Depressive features were assessed using the Patient Health Questionnaire–9 [23], a widely used and validated measure of depressive symptom severity, where higher scores indicate more severe depressive features (range 0-27).

#### Patient View of Progress and Satisfaction

The program’s final session included 2 questions assessing patients’ perceived improvement in their eating problem and understanding of it, as well as a brief satisfaction questionnaire with both closed- and open-ended questions. The questionnaire included selected items adapted from the Client Satisfaction Questionnaire for internet-based interventions [24], alongside program-specific items assessing helpfulness, ease of use, likelihood of recommending the program to others, and perceived ability to live the life they wanted after completion. Open-ended questions invited feedback on what patients liked most and least about the program. This brief questionnaire was used to capture program-specific feedback not fully covered by the standardized outcome measures while keeping response burden low. The questionnaire is provided in Multimedia Appendix 2.

#### Data Analysis

Descriptive statistics were calculated for quantitative measures (means and SDs for normally distributed data, medians and ranges for nonnormally distributed data, and frequencies and percentages for categorical data). Preprogram and postprogram scores were compared using paired 2-tailed *t* tests. Postprogram outcome data were collected in session 12; therefore, clinical outcome analyses were conducted on a completer-only basis.

### Qualitative Methods

#### Data Collection

Semistructured interviews were conducted to explore patients’ experiences of the supported program-led treatment. Interviews were conducted within the evaluation period and scheduled as close as possible to program completion. All patients were offered an optional interview, with the aim of capturing a range of experiences, including those who completed the program and those who did not. However, only patients who completed or were close to completing the program self-selected for an interview.

Patients were initially informed about the evaluation by their supporter. Those who expressed interest and consented to be contacted had their details shared with the independent evaluation team, which then provided further information and arranged interviews. Interviews were conducted by members of the independent evaluation team who were experienced in qualitative research.

An interview guide was used and developed collaboratively by the independent evaluation service, the NHS eating disorder service, the Centre for Research on Eating Disorders at Oxford (CREDO), and Credo Therapies. The guide was informed by the framework for measuring the implementation of behavioral intervention technologies [25] and covered key domains, including acceptability, appropriateness, feasibility, usability, engagement, and perceived impact of the program. Interviews were conducted via Microsoft Teams, lasted approximately 1 hour, and were audio-recorded and transcribed verbatim. Transcripts were imported into NVivo (version 14, Lumivero) for analysis.

To complement the patient interviews and explore reasons for noncompletion, a joint interview was conducted with the 2 MHWPs who delivered the support. This interview followed a similar semistructured format and focused on the supporters’ experiences of supporting patients using the program and their perspectives on patient engagement and dropout.

In addition, NHS staff involved in the pilot (including clinical leads and therapists involved in implementation and supervision) completed a qualitative survey to gather their perspectives on the program and its impact on patients, their roles, and the eating disorder service. The survey, developed by the independent evaluation service in collaboration with project stakeholders, included 9 open-ended questions and was distributed via Microsoft Forms by evaluation leads within the NHS eating disorder service.

#### Data Analysis

Data from the interviews and survey were analyzed together, given the high degree of convergence across patients and staff. Reflexive thematic analysis, following the 6-phase approach described by Braun and Clarke [26], was used to analyze the data. Two members of the independent evaluation team independently coded a subset of transcripts and met to compare interpretations and develop an initial coding framework, which was then applied to subsequent transcripts, with regular discussions within the analysis team to refine codes and develop themes. The initial coding framework was also discussed with collaborators at CREDO to support interpretation and ensure relevance to the service context.

Free-text survey responses were exported into NVivo (version 14) and coded using the same framework developed for the patient interview data, due to the high level of consensus on the themes. Following this, a lived experience contributor at CREDO reviewed the thematic map and theme labels and advised on wording to support the clarity, sensitivity, and accessibility of the findings.

### Integration of Quantitative and Qualitative Findings

Following separate quantitative and qualitative analyses, the findings were integrated using a joint display organized around the evaluation aims (implementation in routine NHS care, treatment uptake and completion, acceptability, and clinical outcomes). Quantitative and qualitative findings were compared to identify areas of convergence and examine how one dataset contextualized the other. Integrated interpretations (meta-inferences) were then developed to explain how the findings collectively addressed the evaluation aims.

## Results

### Suitability and Completion Rates

**Figure 1:**
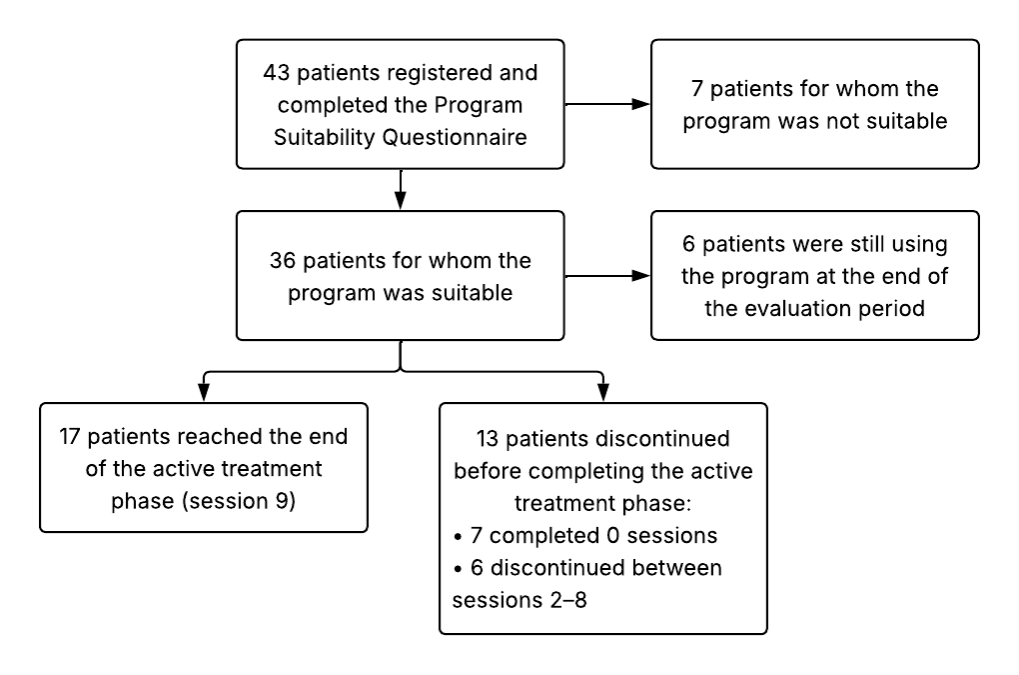
Patient flow through the digital program.

During the evaluation period, 43 patients registered for the program, all of whom completed the Program Suitability Questionnaire (Figure 1). Based on the criteria defined by the NHS eating disorder service, the program was considered suitable for 36 patients (84%). For the remaining 7 patients, the program was deemed unsuitable. Reasons for unsuitability included features suggestive of severe depression or active suicidal ideation or self-harm (n=3), the absence of recurrent binge eating (n=1), the use of compensatory behaviors such as vomiting or misuse of laxatives inconsistent with a diagnosis of BED (n=3), or a current medical or physical condition that influenced or was influenced by day-to-day eating habits (n=2).

Among the 36 patients for whom the program was deemed suitable, 6 were still using the program at the end of the evaluation period. The remaining 30 patients had either completed or discontinued the program. Overall, these patients completed a mean of 7.8 (5.2) sessions (median 12, IQR 2.5-12). Engagement decreased over time: 20/30 patients completed at least 6 sessions, 17/30 reached the end of the active treatment phase (up to and including session 9), and 16/30 completed the full program, including the final sessions focused on maintenance (staying well in the longer term).

Thirteen patients discontinued the program before completing the active treatment phase. Among these, attrition occurred predominantly early, with more than half (7/13) not completing any sessions and the remainder discontinuing between sessions 2 and 8 (mean 2.5, SD 3.1; median 0, IQR 0-5).

### Preprogram Patient Characteristics

Preprogram demographic and clinical characteristics are presented in Table 1. A descriptive comparison of preprogram characteristics between patients who completed all 12 sessions and the postprogram assessment and those who did not is provided in Table 2.

**Table 1:** Preprogram patient characteristics. Clinical characteristics were available for 32 of the 36 patients for whom the program was deemed suitable; 4 patients discontinued before completing the preprogram assessment.

Characteristic			Value
**Demographics^a^** **(n=36)**			
	Age (years), mean (SD; range)	38 (11; 21-66)	
	Female, n (%)	24 (67)	
	Male, n (%)	12 (33)	
	White, n (%)	34 (94)	
	Black, n (%)	2 (6)	
	Years since onset of eating problem, mean (SD; range)	23 (14; 0-50)	
**Clinical characteristics (n=32)**			
	Frequency of objective binge eating (over the past four weeks), mean (SD; range)	16.56 (8.80; 4-50)	
	Eating disorder psychopathology, mean (SD; range)	4.03 (1.11; 1.05-5.80)	
	Secondary impairment, mean (SD; range)	32.19 (7.99; 13–46)	
	Depressive features, mean (SD; range)	12.88 (5.49; 2–24)	

^a^Other gender and ethnicity categories were available but not selected by any patients.

**Table 2:** Preprogram characteristics of patients who completed and did not complete all 12 sessions and the postprogram assessment. Clinical characteristics were available for 10 of the 14 patients who did not complete all 12 sessions and the postprogram assessment; 4 patients discontinued before completing the preprogram assessment.

Characteristic		Completed all 12 sessions and the postprogram assessment (n=16)	Did not complete all 12 sessions and the postprogram assessment (n=14)
**Demographics^a^**			
	Age (years), mean (SD; range)	43 (12; 28-66)	37 (9; 24-51)
	Female, n (%)	14 (88)	7 (50)
	Male, n (%)	2 (13)	7 (50)
	White, n (%)	16 (100)	12 (86)
	Black, n (%)	0 (0)	2 (14)
	Years since onset of eating problem, mean (SD; range)	29 (13; 3-50)	20 (15; 0-50)
**Clinical characteristics**			
	Frequency of objective binge eating (over the past 4 weeks), mean (SD; range)	18.06 (11.28; 4-50)	16.50 (5.44; 6-26)
	Eating disorder psychopathology, mean (SD; range)	3.94 (1.20; 1.05-5.60)	3.98 (1.10; 2.29-5.80)
	Secondary impairment, mean (SD; range)	32.25 (8.87; 13-45)	31.70 (6.68; 14-39)
	Depressive features, mean (SD; range)	12.13 (5.95; 2-24)	13.20 (5.35; 4-20)

^a^Other gender and ethnicity categories were available but not selected by any patients.

### Clinical Outcomes

Patients who completed all 12 sessions and the postprogram assessment reported significant reductions in the frequency of objective binge eating, eating disorder psychopathology, secondary impairment, and features of depression (Table 3). Across measures, higher scores indicate greater severity or impairment.

In addition to these changes in scores, clinically meaningful improvements were also observed in the following areas:

- Frequency of objective binge eating: at the end of the program, 31% (5/16) of patients reported zero objective binge eating episodes in the past 28 days, indicating abstinence from binge eating during this period.
- Eating disorder psychopathology: at preprogram, 19% (3/16) of patients scored below the clinical cutoff of 2.77 [27]. At postprogram, this had increased to 63% (10/16) of patients, indicating a meaningful reduction in eating disorder psychopathology.
- Secondary impairment: at preprogram, only 6% (1/16) of patients scored below the clinical cutoff of 16 [28]. By the end of the program, this had increased to 75% (12/16) of patients, reflecting a 69% improvement in those no longer meeting the threshold for clinically significant impairment.
- Features of depression: the number of patients scoring below the clinical cutoff of 10 [29] (indicating the absence of clinical depression) rose from 31% (5/16) at preprogram to 93% (15/16) at postprogram.

**Table 3:** Comparison of preprogram and postprogram scores for patients completing all 12 sessions and the postprogram assessment (n=16).

Measure	Preprogram, mean (SD)	Postprogram, mean (SD)	*t* test^a^ (*df*; 95% CI)	*P* value
Frequency of objective binge eating (over the past 4 weeks)	18.91 (11.28)	1.88 (1.82)	−6.01 (15; −21.93 to −10.44)	<.001
Eating disorder psychopathology	3.94 (1.20)	1.85 (1.14)	−8.34 (15; −2.62 to −1.55)	<.001
Secondary impairment	30.75 (8.87)	12.56 (9.50)	−10.02 (15; −22.06 to −14.31)	<.001
Features of depression	12.12 (5.95)	4.81 (4.10)	−7.57 (15; −9.37 to −5.26)	<.001

^a^The *t* tests were 2-tailed.

### Perceived Improvement in Eating Problem and Understanding

All patients who completed the postprogram assessment (n=16) reported improvements in their eating problem. A total of 9 described it as “much better,” and 7 as “somewhat better.” Similarly, all 16 patients indicated an improved understanding of their eating problem, with 10 reporting it as “much better” and 6 as “somewhat better.”

### Patient Satisfaction

Almost all (15/16, 94%) patients found the program helpful, with 11/16 rating it as “very helpful” and 4/16 as “moderately helpful.” Similarly, 94% (15/16) found the program easy to use, with 10/16 rating it as “very easy to use” and 5/16 rating it as “moderately easy to use.” When asked whether they would recommend the program to someone with experience of binge eating, patients gave a mean score of 8.8 (SD 2.5) on a scale ranging from 0 (not at all likely) to 10 (extremely likely). Notably, almost all (15/16, 94%) patients reported feeling more able to live the life they wanted after completing the program.

The most commonly reported “most-liked” elements of the program, based on open-ended responses, were its ease of use, access to helpful resources and materials, and the support calls. Reported “least-liked” aspects were technical and functional limitations, perceptions that the program pace was too fast, and concerns that some sections were insufficiently detailed or tailored.

### Qualitative Analysis

A total of 8 patients who had completed or were close to completing the program were interviewed about their experience of the supported program-led treatment. These patients were predominantly female, all identified as White British, and represented age brackets ranging from 26 to 35 years and from 65 to 74 years. Two supporters participated in a joint interview about their experience of the pilot, and 6 staff members (6/10, 60%) completed a survey about their involvement.

**Figure 2:**
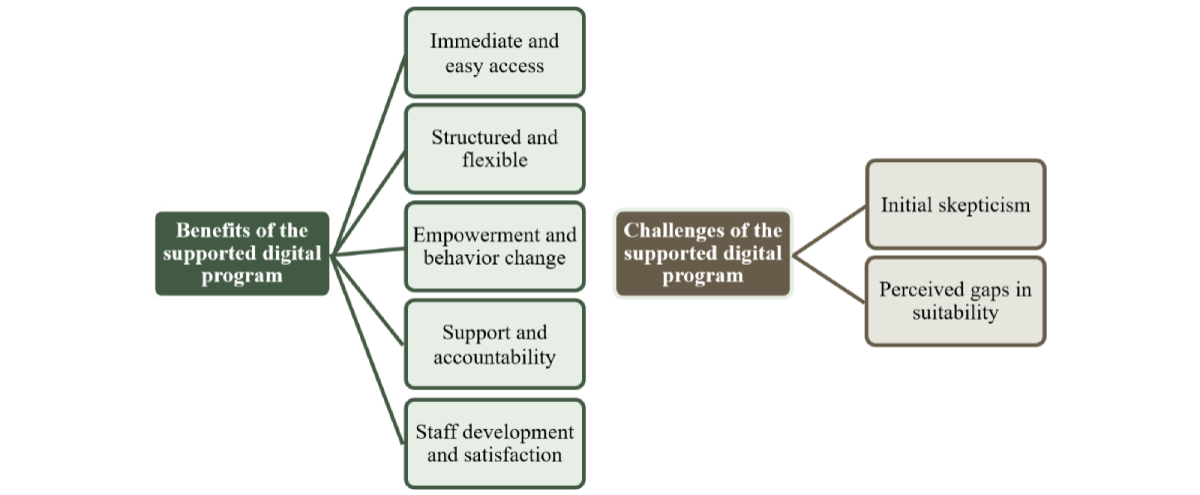
Thematic map.

Data from all interviews and surveys were analyzed together. Staff responses, including those from the supporter interview, are presented as Staff Respondents 1-8. In collaboration with CREDO, the data were organized into 2 overarching themes: benefits of the supported digital program and challenges of the supported digital program (Figure 2).

### Benefits of the Supported Digital Program

#### Immediate and Easy Access

Patients emphasized both the speed and the simplicity of accessing the supported digital program. They described receiving the intervention quickly and with minimal steps: “It all seemed to happen very quickly, which surprised me...I thought there might be quite a long waiting list, and it would take longer” (Patient 6). Clinicians similarly noted faster access compared with usual care: “quicker than what we might see with treatment as usual” (Staff 5). Patients also highlighted the ease of registration, describing it as “straightforward,” “easy,” and that they were “up and running very quickly” (Patient 6).

#### Structured and Flexible

Patients and staff valued the program’s clear structure and ease of use: “I think that it is a well-structured and a seemingly simple app to use” (Staff 1). Alongside this, the flexibility of remote delivery was particularly appreciated, enabling sessions to fit around work and home commitments: “to be able to...do them remotely and work around when I’m at home or in the office...was very, very useful” (Patient 8). Some patients also welcomed the independence this flexibility provided: “if I can have something that I can sort of do independently at home, I prefer that” (Patient 4).

#### Empowerment and Behavior Change

The program helped patients better understand their binge eating, with staff noting that the content supported individuals “to understand what is happening when they binge” (Staff 3). Support calls also provided opportunities to “really problem solve it together” (Staff 7). Patients reported that discussing the ideas made them more usable in practice: “Instead of it just being me reading a piece of paper...discussing it...made it a lot more accessible” (Patient 1). This suggests that discussing the content with a supporter helped patients engage more actively with the material and apply it in practice, supporting a more active role in their recovery. This was reflected in reported behavior changes. Patients described establishing greater routine and consistency—“I think it’s encouraged me to prioritise consistency” (Patient 8)—and reduced binge eating: “...I haven’t binge eaten for weeks” (Patient 2). Others highlighted a sense of responsibility for maintaining progress: “I must keep doing what I've learned and keep practising what I’ve learned to be able to continue to move forward, so I still have work to do” (Patient 7).

#### Support and Accountability

Patients and staff valued the support sessions as a safe, nonjudgmental space to discuss difficult feelings: “an open and non-judgemental space” (Staff 5). The opportunity for regular contact provided reassurance and a counseling-like outlet: “knowing that you’ve got a weekly session with someone as well that you can talk to, so that kind of counselling side to it as well” (Patient 6). Supporters’ listening and reframing helped patients gain confidence in making changes: airing concerns and receiving a response “really helped me...to actually think...I could make a change here” (Interviewee 2). Support also encouraged patients to keep trying when motivation was low: having someone “willing to do something with you is very valuable for lifting you up and making you more willing to sort of try because it's not easy” (Interviewee 3).

Regular check-ins created a sense of accountability, with patients describing how “someone was checking what I would do,” which kept them “on track” and “accountable” (Patient 7). Staff observed the same effect, noting that sessions acted as reminders: “people would quite often do the session like just before I check in. And I think it’s almost like a reminder of, oh, I need to do this” (Staff 7). Some patients felt they would not have continued without this support: “If it hadn’t been for her, I wouldn’t have stuck with it” (Patient 3); “If I just had the digital side, I think I’d have...given up on it” (Patient 7).

#### Staff Development and Satisfaction

One staff member reported that reduced contact time meant they felt able to manage more patients within their caseload: “I am able to manage more patients under my caseload as the contact time is significantly reduced compared to treatment as usual” (Staff 3). This increased efficiency was seen as part of a wider service transformation, with digital delivery “increasing the digital footfall of the service,” supporting “our transformation agenda” and “highlight[ing] this developing area of my role” (Staff 2). Staff also reported feeling more organized, confident, and satisfied in their work: “[I] feel that I have a set place in the service now, it has helped me to gain confidence in my abilities to support patients” (Staff 5).

### Challenges of the Supported Digital Program

#### Initial Skepticism

Some patients described early skepticism of the digital format, admitting that they “thought...I wasn’t going to get anything at all from it being online” (Patient 1). However, this view shifted after the program, such that “having done it now..., it’s opened my eyes a bit and it’s really helped me learn” (Patient 8), with the support calls offering a good balance of contact and independence: “has been really good having the combination of both” (Patient 8).

#### Perceived Gaps in Suitability

Staff highlighted that the supported digital program may not be suitable for all patients, particularly those experiencing significant life stressors or co-occurring conditions. One staff member explained that some patients “don’t really have the space to be motivated and engaged with the programme” due to “other mental health problems...or personal stresses” (Staff 7). Others noted that individuals with more complex presentations may require more intensive support than the program can offer:

It may not provide sufficient support for those patients who have other comorbid difficulties and therefore have more complex presentation e.g. low mood.

Staff 1

This was seen as limiting its applicability:

A lot of people… will often have low mood and that does really shrink the amount of people that can do [the digital programme] and get a lot of benefits out of it.

Staff 7

A small number of patients also felt the support calls ended too soon, leaving them without enough time to consolidate changes:

I need more support… you’re halfway through changing your like 25 years of this, and then it's just cut off within 12 weeks and… like I’m back on my own again… and…my eating has gone back. Really far back.

Patient 5

This led the patient to suggest a follow-up support call around a month after the end of the program to ease the transition.

### Integrated Findings

Quantitative and qualitative findings were integrated using a joint display organized around the evaluation aims (Table 4).

**Table 4:** Integration of quantitative and qualitative findings across the evaluation aims.

Evaluation aim	Quantitative finding	Qualitative finding	Integrated interpretation
Implementation in routine NHS^a^ care	The program was deemed suitable for 84% (36/43) of the registered patients.	Staff described the intervention as feasible to integrate into routine care and suggested it could reduce clinician contact time per patient.	Quantitative and qualitative findings converged to suggest that the program could be integrated into an existing NHS eating disorder pathway and may be suitable for use with a substantial proportion of patients presenting with BED^b^.
Treatment uptake and completion	At the time of the evaluation, 6 patients were still using the program, 17 completed the active treatment phase, and 13 discontinued.	Staff identified low motivation, competing life demands, and co-occurring difficulties as barriers to engagement. Some patients reported initial skepticism about digital treatment.	Qualitative findings helped contextualize the observed pattern of uptake, early discontinuation, and completion, suggesting that engagement may be influenced by motivation, competing demands, co-occurring difficulties, and initial skepticism.
Acceptability to patients and staff	Among patients who completed the program, satisfaction ratings were high, and most reported finding the program helpful and easy to use.	Patients valued the program’s accessibility, flexibility, support, and accountability, while staff reported increased confidence, work satisfaction, and perceived service benefits.	Quantitative and qualitative findings were consistent in suggesting that the supported digital program was acceptable to patients who completed the program and to NHS staff involved in delivery.
Clinical outcomes	Patients who completed the program reported significant reductions in the frequency of binge eating, eating disorder symptoms, depressive features, and secondary impairment.	Patients described perceived improvements in their binge eating and understanding of it, greater routine and consistency in their eating, and a sense of responsibility for maintaining progress.	Qualitative accounts of behavior change and improved understanding were consistent with the observed quantitative improvements among patients who completed the program.

^a^NHS: National Health Service.

^b^BED: binge eating disorder.

## Discussion

### Principal Findings

This service improvement project evaluated the real-world implementation of the supported digital program-led version of CBT-E for adults with BED within an NHS specialist eating disorder service. Quantitative and qualitative findings were broadly consistent, suggesting that the intervention could be implemented in routine care, was acceptable to patients and staff, and was associated with meaningful uptake, completion, and preliminary improvements in clinical outcomes among those who completed the program.

Implementation feasibility was supported by uptake and completion data. Over 10 months, 43 patients registered, and the program was considered suitable for 36 (84%). At the time of the evaluation, 6 patients were still using the program, while 30 had either completed or discontinued the program. Of these, 17 reached the end of the active treatment phase, and 16 completed all sessions and the postprogram assessment. Among these 16 patients, significant reductions were observed in the frequency of objective binge eating, eating disorder psychopathology, associated impairment, and depressive features. These changes were characterized by large effect sizes, with most patients scoring below established clinical cutoffs at the end of the program. These improvements were accompanied by high levels of patient-reported satisfaction and perceived benefit among those who completed the program, with most patients rating the program as helpful and easy to use, reporting an improved understanding of their eating problem, indicating that they would recommend it to others, and nearly all reporting feeling more able to live the life they wanted following treatment.

However, it is important to note that 13 (43%) patients did not complete the program. Discontinuation occurred predominantly early in the program, with more than half of those who discontinued not completing any sessions, suggesting that barriers to engagement may arise before or shortly after treatment initiation. Due to limited data on patients who did not complete the program, it is not possible to draw firm conclusions regarding the reasons for discontinuation. However, this attrition highlights the need for further investigation into barriers to engagement and completion.

These findings were reinforced by qualitative accounts highlighting the program’s accessibility, structured yet flexible format, and the value of remote support sessions in fostering accountability and motivation. Staff described the intervention as feasible to integrate into routine care and suggested that it could reduce clinician contact time per patient, supporting more efficient service delivery. They also reported increased satisfaction. Qualitative findings helped contextualize patterns of uptake and completion. Some patients described initial skepticism about digital delivery, while staff suggested that low motivation, competing life demands, and co-occurring difficulties could make engagement more challenging for some individuals. Together, these findings may help explain why discontinuation occurred predominantly early in the program, although further research is needed to better understand the reasons for noncompletion. Overall, this approach appears to be acceptable and may improve access to evidence-based treatment for binge eating within routine NHS care.

### Interpretation and Comparison With Existing Literature

This evaluation supports the NHS commitment to expanding digital provision in mental health care [15] and contributes real-world evidence to the literature on digital interventions for eating disorders. Outcomes among patients who completed the program in this service improvement context are broadly consistent with those reported in other evaluations of digital or virtual GSH for binge eating. For example, a recent service evaluation of virtually delivered GSH via videoconferencing and a cognitive behavioral self-help book also reported large reductions in binge eating frequency among patients who completed the program [14]. Completion rates were also similar, with 60% completing treatment in that evaluation [14], compared with 57% (17/30) completing all active treatment sessions in the present evaluation.

In contrast, completion rates in the present evaluation were lower than those reported in research trials conducted under more tightly controlled conditions, such as an internet-based GSH trial for BED in which 78% completed treatment [30]. It is generally expected that higher completion rates are associated with better outcomes, and evidence suggests that engagement has a small but significant association with clinical improvement [31]. However, because temporal precedence was not established in that study, it is also possible that early improvement may have driven adherence rather than the reverse. This discrepancy in completion rates may reflect differences between controlled research conditions and routine clinical settings, including greater researcher contact, structured follow-up, and compensation in trials. Meta-analytic evidence supports this interpretation, showing higher attrition when studies lack compensation, engagement reminders, or direct researcher contact at enrollment [32]. Differences in the populations studied may also contribute, as specialist services often treat individuals with greater severity, chronicity, and complexity than those typically included in research trials. Consistent with this, patients in the present evaluation reported that their eating problem had started an average of 23 years earlier, suggesting that many had experienced longstanding difficulties before accessing the intervention.

Notably, completion in this evaluation (17/30, 57%) exceeded that observed in another NHS service evaluation of the same digital intervention delivered without support (38%) [33], aligning with evidence that guidance improves engagement in digital interventions [34]. The inclusion of brief, structured support in the current approach may therefore enhance engagement while requiring relatively minimal professional input.

These findings are consistent with stepped-care models. NICE recommends this approach to treating adults with BED, with a BED-focused GSH program offered as a first-line intervention followed by more intensive therapist-led CBT-ED where needed [7]. This is consistent with the present findings, where the program was not considered suitable for some patients, and others did not complete it, indicating the need for flexible progression within the care pathway.

This approach may also help address implementation challenges in routine care, for example, by reducing the burden on specialist staff, as support can be delivered by nonspecialists and is brief in duration. In practice, this may enable specialist services to direct limited clinician time toward patients requiring more intensive support while still increasing access to evidence-based treatment for binge eating. As part of this service improvement, staff received training and model-specific supervision, which may have supported consistent delivery and confidence, although fidelity was not formally assessed. Future work should examine whether similar outcomes can be achieved without additional supervision to determine the requirements for sustainable implementation at scale.

Qualitative findings highlighted the importance of the support calls in fostering accountability, motivation, and connection. This contrasts with feedback from the same intervention delivered without support [33], where patients reported a desire for human contact. Brief supportive calls were considered helpful here for clarifying concepts, normalizing difficulties, and supporting reflection. This is consistent with recent work suggesting that the role of the guide in GSH is to support adherence, personalize the intervention, and maintain motivation through a combination of support and accountability [35].

However, initial skepticism about digital delivery remained a barrier for some patients. This suggests that how such interventions are introduced may influence engagement. Future implementation should monitor reasons for skepticism and address these concerns more proactively, for example, by emphasizing the potential benefits of these interventions and incorporating lived experience perspectives to enhance credibility and hope. Consistent with this, qualitative evidence from people with lived experience of binge eating and health care professionals indicates that compassionate, hopeful education can enhance uptake and engagement with digital interventions for binge eating [36].

### Limitations

This evaluation has several limitations. The relatively small number of patients, together with the absence of a formal power calculation, limits the precision of the estimates. As a service improvement evaluation conducted within a single NHS service, the findings were not intended to be generalized, although they may be informative for similar services and contexts. The patients were also predominantly White, limiting applicability to more diverse populations and highlighting the need to improve equity of access.

Outcome data were available only for patients who completed the program, so it remains unclear whether those who discontinued experienced similar benefits or whether dropout was related to limited clinical response. While the descriptive comparison of patients who did and did not complete all sessions (Table 2) should be interpreted with caution, given the small numbers involved, a greater proportion of male patients and the only 2 Black patients in the evaluation were represented among those who did not complete all sessions, highlighting the importance of future work examining engagement and completion across diverse groups. Future work should aim to collect data from those who did not complete the program to better understand treatment effects and barriers to engagement. The absence of a control group also limits causal inference.

As the evaluation was conducted within routine clinical care, access to the program was determined by existing referral pathways, which may have constrained reach. Patient satisfaction data were only available from patients who completed the program and are therefore likely to overestimate acceptability. In addition, satisfaction was assessed using a brief, nonvalidated questionnaire, and the findings should therefore be interpreted with caution.

Finally, several authors have commercial affiliations with the intervention, introducing the potential for researcher allegiance bias. However, the use of an independent evaluation service to conduct clinical analyses and qualitative data collection and analysis aimed to mitigate this risk. Despite these limitations, the findings provide useful real-world evidence to inform future implementation.

### Implications for Practice and Future Research

This evaluation demonstrates how digital delivery can support the translation of evidence-based interventions into scalable service models within NHS specialist services. Supported digital program-led CBT-E offers an accessible and acceptable treatment option for adults with BED, with brief support enhancing engagement while requiring minimal professional input. Staff and patients perceived potential benefits for service efficiency and access, although waiting list effects were not assessed. By addressing common service- and patient-level barriers, such as limited staff capacity, variable clinician confidence, and patient shame surrounding binge eating, this approach may support the implementation of first-line treatment within routine care.

Future work should examine reasons for discontinuation, outcomes among patients who did not complete the program, and predictors of engagement and response. More work is also needed to determine how best to adapt the intervention for individuals with low motivation or co-occurring conditions and how to provide effective follow-up support. Controlled evaluations comparing this approach with treatment as usual are required to establish effectiveness. Health economic analyses are needed to assess cost-effectiveness and potential resource savings. In addition, future work should prioritize improving equity of access and evaluating the long-term maintenance of treatment gains.

### Conclusion

This evaluation suggests that supported digital program-led CBT-E may increase access to NICE-recommended first-line treatment for binge eating within routine care. As a scalable approach, it has the potential to reduce pressure on specialist services while improving treatment reach. Continued evaluation of and investment in digitally enabled models of care will be important to support their sustainable integration into clinical practice.

## Appendix

Multimedia Appendix 1 Modifications to the Eating Disorder Examination–Questionnaire in the digital programme.

Multimedia Appendix 2 Satisfaction Questionnaire.

## Abbreviations

BED: binge eating disorder
CBT-E: enhanced cognitive behavior therapy
CBT-ED: eating-disorder–focused cognitive behavioral therapy
CREDO: Centre for Research on Eating Disorders at Oxford
EDE-Q: Eating Disorder Examination–Questionnaire
GSH: guided self-help
MHWP: mental health and well-being practitioner
NHS: National Health Service
NICE: National Institute for Health and Care Excellence

## Data Availability

The datasets generated during this project are not publicly available due to privacy protection concerns.
