# Supplementary material for: Implementing Supported Digital Enhanced Cognitive Behavior Therapy for Binge Eating Disorder in Routine Care: Mixed Methods Service Evaluation
Source: J Med Internet Res. 2026 Jul 17;28:e92069. doi: 10.2196/92069 (PMC13428200; doi:10.2196/92069)
Supplement: Multimedia Appendix 1 [file jmir_v28i1e92069_app1.docx]

**Article title**: Implementing Supported Digital Enhanced Cognitive Behavior Therapy for Binge Eating Disorder in Routine Care: Mixed Methods Service Evaluation

**Journal name**: *Journal of Medical Internet Research*

**Author names**: Osborne, E. L., Powell, J., Brown, C., Cresswell-Nash, G., Debrou, L., Defever, E., Greenwood, S., Horton, J., Hunter, E., Lees, A., Moore, M., Newell, C., Randell, B., Rosten, C., Shaw, N., Yao, V., & Murphy, R.

**Corresponding author**: Emma L. Osborne, Centre for Research on Eating Disorders at Oxford, Department of Psychiatry, University of Oxford, Warneford Hospital, Oxford, OX3 7JX, UK, emma.osborne@psych.ox.ac.uk

# Multimedia Appendix 1: Modifications to the Eating Disorder Examination–Questionnaire in the Digital Programme

The modification to the Eating Disorder Examination–Questionnaire (EDE-Q; Fairburn & Beglin, 2008) in the digital programme does not affect calculation of the EDE-Q global and subscale scores. The wording of questions 13, 14, and 15 in the EDE-Q in the digital programme have been modified. The reason for this is to include a measure of subjective binge eating, which refers to episodes of loss of control over eating when the amount of food consumed is not objectively large. The original EDE-Q does not tell us about subjective binge eating; however, the modifications to questions 13–15 tell us about frequency of subjective binge eating without additional burden.

**References**

Fairburn, C. G., & Beglin, S. J. (1994). Assessment of eating disorders: Interview or self-report questionnaire? *The International journal of eating disorders*, *16*(4), 363–370. https://doi.org/10.1002/1098-108X(199412)16:4%3C363::AID-EAT2260160405%3E3.0.CO;2-%23
