# Supplementary material for: Implementing Supported Digital Enhanced Cognitive Behavior Therapy for Binge Eating Disorder in Routine Care: Mixed Methods Service Evaluation
Source: J Med Internet Res. 2026 Jul 17;28:e92069. doi: 10.2196/92069 (PMC13428200; doi:10.2196/92069)
Supplement: Multimedia Appendix 2 [file jmir_v28i1e92069_app2.docx]

**Article title**: Implementing Supported Digital Enhanced Cognitive Behavior Therapy for Binge Eating Disorder in Routine Care: Mixed Methods Service Evaluation

**Journal name**: *Journal of Medical Internet Research*

**Author names**: Osborne, E. L., Powell, J., Brown, C., Cresswell-Nash, G., Debrou, L., Defever, E., Greenwood, S., Horton, J., Hunter, E., Lees, A., Moore, M., Newell, C., Randell, B., Rosten, C., Shaw, N., Yao, V., & Murphy, R.

**Corresponding author**: Emma L. Osborne, Centre for Research on Eating Disorders at Oxford, Department of Psychiatry, University of Oxford, Warneford Hospital, Oxford, OX3 7JX, UK, emma.osborne@psych.ox.ac.uk

# Multimedia Appendix 2: Satisfaction Questionnaire

1. Did you find the digital programme helpful?

Not at all helpful, somewhat helpful, moderately helpful, very helpful

1. Did you find the digital programme easy to use?

Not at all easy to use, somewhat easy to use, moderately easy to use, very easy to use

1. Did you find the SMS nudges (if applicable) helpful?

Not at all helpful, somewhat helpful, moderately helpful, very helpful

1. What did you think about the duration of the digital programme?

The digital programme is too short – 8 weeks isn’t enough

The digital programme is about the right length – 8 weeks seems about right

The digital programme is too long – 8 weeks is too long

1. Would you recommend the digital programme to someone else who has a binge eating problem?

0 – not at all likely, 1, 2, 3, 4, 5, 6, 7, 8, 9, 10 – Extremely likely

1. Was there anything you particularly liked about the digital programme?

[Free text box]

1. Was there anything you particularly disliked about the digital programme?

[Free text box]

1. After completing the digital programme, do you feel more able to live the life you want to live?

Yes, no
